# Increased neurocardiological interplay after mindfulness meditation: a brain oscillation-based approach

**DOI:** 10.3389/fnhum.2023.1008490

**Published:** 2023-06-19

**Authors:** Junling Gao, Rui Sun, Hang Kin Leung, Adam Roberts, Bonnie Wai Yan Wu, Eric W. Tsang, Andrew C. W. Tang, Hin Hung Sik

**Affiliations:** ^1^Buddhist Practices and Counselling Science Lab, Centre of Buddhist Studies, The University of Hong Kong, Hong Kong, Hong Kong SAR, China; ^2^Department of Rehabilitation Sciences, The Hong Kong Polytechnic University, Hong Kong, Hong Kong SAR, China; ^3^Singapore-ETH Centre, Future Resilient Systems Programme, Singapore, Singapore; ^4^Department of Psychology, HKU School of Professional and Continuing Education, Hong Kong, Hong Kong SAR, China

**Keywords:** alpha peak frequency, mindfulness meditation, heart coherence, brain-heart connection, effective connectivity, resting-state EEG

## Abstract

**Background:**

Brain oscillations facilitate interaction within the brain network and between the brain and heart activities, and the alpha wave, as a prominent brain oscillation, plays a major role in these coherent activities. We hypothesize that mindfully breathing can make the brain and heart activities more coherent in terms of increased connectivity between the electroencephalogram (EEG) and electrocardiogram (ECG) signals.

**Methods:**

Eleven participants (28–52 years) attended 8 weeks of Mindfulness Based Stress Reduction (MBSR) training. EEG and ECG data of two states of mindful breathing and rest, both eye-closed, were recorded before and after the training. EEGLAB was used to analyze the alpha band (8–12 Hz) power, alpha peak frequency (APF), peak power and coherence. FMRIB toolbox was used to extract the ECG data. Heart coherence (HC) and heartbeat evoked potential (HEP) were calculated for further correlation analysis.

**Results:**

After 8 weeks of MBSR training, the correlation between APF and HC increased significantly in the middle frontal region and bilateral temporal regions. The correlation between alpha coherence and heart coherence had similar changes, while alpha peak power did not reflect such changes. In contrast, spectrum analysis alone did not show difference before and after MBSR training.

**Conclusion:**

The brain works in rhythmic oscillation, and this rhythmic connection becomes more coherent with cardiac activity after 8 weeks of MBSR training. Individual APF is relatively stable and its interplay with cardiac activity may be a more sensitive index than power spectrum by monitoring the brain-heart connection. This preliminary study has important implications for the neuroscientific measurement of meditative practice.

## 1. Introduction

Mindfulness meditation, a popular form of meditation, has demonstrated a wide range of benefits across various domains such as education, clinical settings, commercial industries, and the military (Goldberg et al., 2020; Duff, 2022). Mind-body connection is central to mindfulness meditation, and recent studies suggest that meditation can modulate brain network organization and the neural representation of cardiac activity within the default mode network (Jiang et al., 2020; Lurz and Ladwig, 2022; Wong et al., 2022). Nevertheless, research on the potential neural mechanisms underlying brain-heart connection remains relatively scarce when compared to enormous research on other mechanisms of mindfulness (Ng et al., 2005; Minhas et al., 2022). Our previous study demonstrated brain-heart entrainment in mindfulness meditation practitioners, however, it only examined the data at a group-level (Gao et al., 2016). To better understand how the brain and body interact during meditation, this study focuses on instantaneous brain-heart entrainment during mindfulness meditation practice at an individual level, which would support a greater application in mindfulness practice.

Naturally, immediate mind-body connections can be felt by individuals in their response to significant events or intense emotions, with the heart being particularly responsive. This is because that central nervous system modulates visceral organ activity through the autonomic nervous system, with most visceral organs functioning autonomously but exhibiting a clear circadian rhythm (Tran et al., 2021; Chambers et al., 2022). Maintaining coherent mind-body activity and circadian rhythms is essential for our well-being, and disruptions can lead to visceral organ dysfunction or even cardiac arrest (Tran et al., 2021). Recognizing the importance of mind-body coherence, the bio-medical-social model has been proposed for promoting health (Heidger, 2011).

To simplify the investigation of the mind-body connection, this study examines the relationship between cerebral and cardiac activities, as the heart is the most responsive organ to external stimuli (Lutwak and Dill, 2012). Electroencephalography (EEG) and electrocardiography (ECG) can easily measure brain and cardiac activities, respectively. Different EEG frequency bands, such as delta, theta, alpha, beta, and gamma, reflect various mental states. Among these, the alpha wave is the primary human brain oscillation, with changes in alpha wave activity being the most reliable outcomes in EEG meditation studies (Lomas et al., 2015).

Different meditation forms can induce changes in distinct brain wave bands; for example, traditional Tibetan Buddhist meditation is associated with gamma band changes (Lutz et al., 2004; Ferrarelli et al., 2013; Jiang et al., 2020). Research has also shown that the anterior cingulate cortex connects to the autonomic nervous system (Devinsky et al., 1995), and frontal midline theta rhythm correlates with heart rate variability during meditation (Kubota et al., 2001). Nonetheless, increased alpha wave activity, particularly in the occipital and frontal regions, has been universally observed during various meditations (Cahn and Polich, 2006).

In this study, we focus on alpha wave analysis due to its significance in brain rhythm and dominance during eyes-closed relaxation which has been proposed as a form of “cortical idling” (Vanwinsum et al., 1984). The alpha power is inversely proportional to the fraction of cortical neurons recruited for any given task (Gevins and Schaffer, 1980). There is similarity of alpha peak frequency (APF) to the central processing unit’s main frequency within the brain network (Bera, 2015). The APF has been implicated in cognitive preparedness (Angelakis et al., 2004) and is positively correlated with processing speed, memory, and cognitive performance in healthy individuals (Vogt et al., 1998; Clark et al., 2004; Moretti et al., 2013; Sanchez-Lopez et al., 2018). Clinical patients typically exhibit reduced APF and lower cognitive performance compared to healthy individuals (Klimesch et al., 1993; Bertaccini et al., 2022). Higher alpha frequency is associated with better academic performance (Klimesch, 1999) and intelligence (Anokhin and Vogel, 1996).

Alpha peak frequency is a largely heritable trait, with heritability accounting for most individual APF (iAPF) variance (Mierau et al., 2017). Although iAPF is highly stable across time and considered a heritable neurophysiological marker and true endophenotype, evidence suggests that APF can be volatile during different mental states and may reflect a change in cerebral oxygenation (Angelakis et al., 2004). Alpha wave activity can be measured by averaging alpha band power within the 8–12 Hz range, and alpha peak power can be specified to calibrate the highest alpha power in 8–12 Hz range. Slight peak power of variations across brain regions may occur due to differences in brain network interactions (Mierau et al., 2017).

To measure the interactive activity among brain network, the topography of this interactive synchronization can be calculated by alpha coherence (Zheng et al., 2007). Coherence is typically calculated using mean cross-power density over respective mean auto-power spectral densities. Different methods have been developed to calculate brain connectivity, and mindfulness training has been shown to modulate brain connectivity in diverse populations (Kilpatrick et al., 2011; Jovanovic et al., 2013; Lifshitz et al., 2019; Li et al., 2022).

Alpha waves are generated by thalamo-cortical information interactions, such as feedback loops between excitatory and inhibitory neurons (Dasilva, 1991; van Schie and Bekkering, 2007). These loops facilitate cortical information processing and communication with the thalamus, potentially causing slight iAPF shifts in different mental states. The interplay between the thalamus and cortex plays a critical role in various brain functions, including consciousness and perception, and may contribute to brain-heart activity interactions (Min et al., 2020).

The dynamic interaction between cerebral and cardiac electrical activities can be directly reflected by the heartbeat evoked potential (HEP). The R-peak of QRS waves in ECG is considered an event related to ongoing cerebral electrical activity, allowing HEP calculation using event-related potential methods. HEP varies among individuals due to differences in body structure and function, with higher HEP observed in those with greater interoception (Jiang et al., 2020; Coll et al., 2021).

This study aims to investigate brain-heart interaction with a focus on alpha oscillation, given its prominence in meditation research. We primarily analyze the oscillation and synchronization of neurophysiological systems between the brain network and cardiac activity. By examining the fundamental synchronization, we would demonstrate the critical role of brain-heart coherence in a meditative state. Additionally, we did coherence analysis in quantitative EEG to provide cortico-cortical interactions and further topographical information on coherent oscillatory activity during mindfulness meditation.

## 2. Materials and methods

### 2.1. Participants

Thirteen healthy participants from a local Mindfulness Based Stress Reduction (MBSR) class were recruited for this EEG study. Each of them was paid $HK200 to compensate for their time. The Beck Depression Inventory (BDI) was used to exclude participants with depression (Yeung et al., 2002). All participants had completed education at or above the undergraduate level. None of them had experience in MBSR. During the experiment, they were requested to only practice the mindfulness breathing technique learned from their MBSR course. The course was taught following the standard MBSR program consisting of one pre-program orientation session, eight weekly classes and one all-day class when the teacher provided direct instructions. Also, participants had to make a strong commitment to practice 45 min of MBSR training each day as home assignments for 8 weeks individually, which included body scanning and mindful breathing. The research was approved by the local Institutional Review Board (IRB) and participants provided their written informed consent before participating in this study. One participant dropped out, and another participant had unusable EEG data due to technical issues. Eventually, data from eleven participants (6 males, 5 females, mean age 35.7 years; 7 Asians and 4 Caucasians) were used for the final data report in this brief research.

### 2.2. Experiment procedure

Electroencephalogram data were recorded first at the beginning of the MBSR training (within 2 weeks), which was taught by a qualified MBSR teacher. The second round of EEG data collection was collected less than 1 month after the MBSR course, resulting in two data sets with two conditions. One condition was having the participants undergo 10 min of eye-closed normal rest, while the other condition was 10 min of eye-closed mindful breathing. Participants were instructed before each task not to ruminate or fall asleep, and the experiment took place in a quiet room. To make sure that trainees knew the correct way to perform the MBSR mindful breathing, the early-stage MBSR condition was set as 2 weeks after of training MBSR. All of the participants confirmed they did follow the instructions after the experiment and their practice of mindfulness breathing was generally good. The Five Facets Mindfulness Questionnaire (FFMQ) was used to measure the change in mindfulness practice quality, and the score of non-reacting facet increased from 22.8 ± 6.5 to 23.9 ± 7.3 (*p* = 0.042). Please refer to the previous paper for more details (Gao et al., 2016). The sequence of rest and mindful breathing was counter-balanced, with half of the participants randomly assigned to perform the mindful breathing task first and the other half doing the rest task first.

### 2.3. Data acquisition and analysis

The data were acquired by a 128-channel NeuroSCAN system in a quiet room. The sampling rate was set at 1,000 Hz. The system has the reference default at the left mastoid, and we recomputed the reference to the combination of both mastoids afterward during data processing. The ECG electrodes were placed at the left and right infraclavicular fossae after cleaning the area with alcohol.

Heart coherence was calculated by peak power divided by total power (McCraty and Zayas, 2014; McCraty, 2022), where peak power was determined by calculating the integral in a window 0.03 Hz wide, centered on the highest peak in the 0.04–0.4 Hz range of the HRV power spectrum, and the total power was determined by calculating the integral in a window of 0.0033–0.4 Hz wide. Heart coherence has a value between 0 and 1 and indicates the magnitude of similarity between the waveform of the HRV tachogram and a sinusoidal wave (McCraty and Zayas, 2014; McCraty, 2022).

EEGLAB was used to extract the power of alpha peak, peak frequency of the alpha wave, and alpha coherence. Eye movement and ECG artifacts in the EEG data were cleaned using independent component analysis (ICA). To evaluate the instantaneous heart and brain coupling of each participant, we found the interval RR from ECG data using the detect function of FMRIB toolbox, then calculated the participant’s heart coherence. Using the RR interval obtained, we set the window length accordingly for further power spectrum analysis of the EEG data. Given the prominence of the alpha wave, this study mainly focused on the alpha wave band (8–12 Hz).

Functional connectivity between brain regions was estimated from EEG coherence between electrodes overlying the participant’s head (Bowyer, 2016). Coherence is one mathematical method used to determine if two or more sensors, or brain regions, have similar neuronal oscillatory activity. Coherence ranges from zero to one, with a value near one indicating that EEG signals have similar phase and amplitude differences at all time points and a value near zero indicating that signals have a random difference in phase and amplitude (Bowyer, 2016). In this study, we used lagged coherence to exclude the non-lagged part of coherence which is believed to be effects of volume conduction (Milz et al., 2014). The lagged coherence calculation details can be calculated as following,


$$L\,a\,g\,C\,o\,h\,\left(f\right)=\frac{{\left[I\,m\,G_{x\,y}\,\left(f\right)\right]}^{2}}{G_{x\,x}\,\left(f\right)\,G_{y\,y}\,\left(f\right)-{\left[R\,e\,G_{x\,y}\,\left(f\right)\right]}^{2}}$$

where *G*_*xy*_(*f*) is the cross-power spectral density and *G*_*xx*_(*f*),*G*_*yy*_(*f*) are the auto-power spectral densities for each channel X and Y.

To calculate the instantaneous brain-heart interplay, a sliding window of 60 s was used, and the Pearson correlation coefficient was calculated between heart coherence and these alpha wave indexes (Kim et al., 2013, 2014). This process returned an *r*-value for each channel and each condition. The *r*-value difference between before and after intervention was then calculated, and finally, paired-sample *t*-tests were used to compare this *r*-value difference between the mindful breathing and rest conditions. Similar indices were calculated for the gamma wave.

Heartbeat evoked potential (HEP) was also calculated, as HEP can reflect the interaction between cardiac and cerebral activities (Jiang et al., 2020; Coll et al., 2021). The HEP was calculated by extracting epochs that were time-locked to the R peaks of QRS wave identified earlier in ECG channel, with the time window set as −200 to 600 ms around the R peak event onset. Then epochs were averaged to obtain the HEP data for each participant. For each channel, paired-sample *t*-tests were used to analyze the difference between conditions. Instantaneous correlation between HEP and alpha wave indexes were also explored. Finally, we also calculated brain effective connectivity with partial directed coherence (PDC) (Baccala and Sameshima, 2001). Significant differences were determined using *p* < 0.05. The flowchart of data collection and analysis is shown in Supplementary Figure 1.

## 3. Results

After MBSR training, participants had a greater brain-heart connection during mindful breathing than that at the early-stage training. This training effect did not happen during close-eye rest.

**FIGURE 1:**
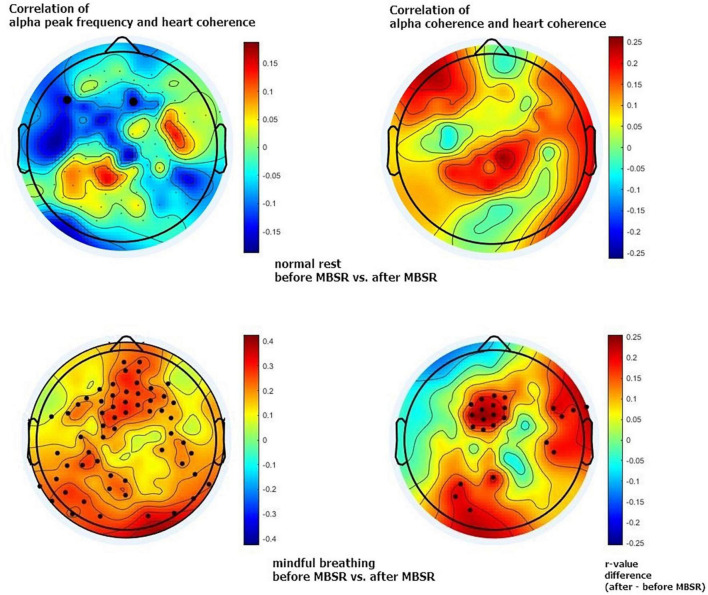
Maps of brain-heart connections. This connection (*r*-value) was calculated based on correlation of alpha peak frequency and heart coherence **(left column)**, and correlation of alpha coherence and heart coherence **(right column)**. The upper row is showing normal rest control condition before and after 8-week mindfulness-based stress reduction (MBSR) training; the lower row is showing mindful breathing condition before and after MBSR training. Color represents the *r*-value differences between before and after MBSR training. The black dots indicate electrodes with significant differences before and after MBSR training (*p* < 0.05, uncorrected).

**FIGURE 2:**
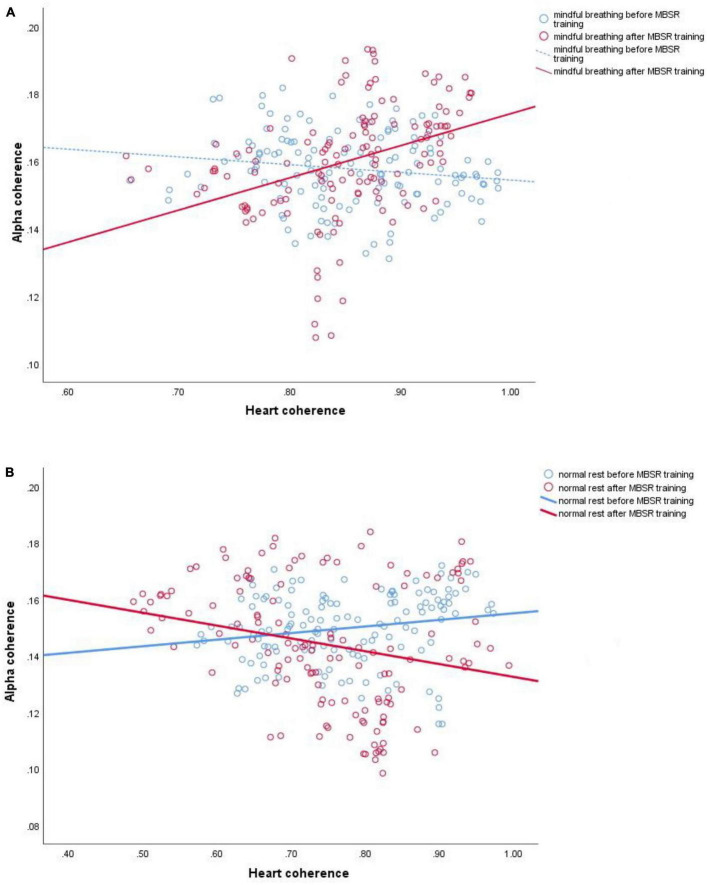
Examples of linear regressions for channel Fz of one participant. **(A)** Instantaneous correlation (60 s, with 4 s sliding) of alpha coherence and heart coherence during mindful breathing before (*r* = –0.153, *p* = 0.077; blue line) and after (*r* = 0.361, *p* < 0.0001; red line) mindfulness-based stress reduction (MBSR) training; **(B)** normal rest before (*r* = 0.190, *p* = 0.028; blue line) and after (*r* = –0.226, *p* = 0.008; red line) MBSR training. Solid line represents significant correlation (*p* < 0.05).

This higher brain-heart connection is most obvious between the alpha peak and heart coherence but also appears between alpha-coherence and heart coherence, with a similar distribution. The increased brain-heart connection is most apparent in the middle frontal and also appears in the temporal and occipital regions of the scalp. See Figures 1, 2.

Figure 1 shows an overall 2-D map of brain areas with increased brain-heart connection after 8-week mindfulness training; while Figure 2 shows a single channel with significantly increased brain-heart connection in terms of alpha coherence and heart coherence. That is, a dark dot means a significant change of brain-heart connection after mindfulness training (see Supplementary Tables 1, 2 for statistic).

Additionally, we calculated heart coherence and gamma coherence, which did not show a significant result. See Supplementary Figure 2. Also, results of HEP analysis did not show any difference after mindfulness training. Correlational analysis between HEP and various alpha indexes were also done. See Supplementary Figures 3–5. The power spectral analysis of alpha power, alpha peak power, alpha peak frequency, and alpha coherence are shown in the Supplementary Figures 6–9, respectively.

With regard to connectivity among brain regions only, these brain connectivity results are shown in the Supplementary Figure 10 and Supplementary Tables 3, 4. It demonstrates significantly increased brain connectivity was found after MBSR training, in terms of PDC indices, while less increased brain connectivity is found during the resting state. The increased brain connectivity was found mainly in the middle frontal with other regions, including the temporal, parietal and occipital regions.

## 4. Discussion

The current study finds that the link between the brain and heart during meditation may follow coherent rhythms rather than amplitudes of neurophysiological activity due to a stronger correlation between heart coherence and alpha wave peak frequency instead of its peak power. Our previous study on mindfulness meditation found entrainment between brain and heart, but only in a group of meditators (Gao et al., 2016). That study did not examine any longitudinal effect of mindfulness meditation either, as only spectrum analysis was made on EEG. Similarly, we did not find significant longitudinal change in MBSR training when directly comparing the spectral analysis of alpha band, including the alpha power, alpha peak power, APF, alpha coherence in the current study. However, we demonstrated that instantaneous brain-heart connection considering both central and peripheral physiological activities can be more sensitive to mental state, and to measure the longitudinal effect of mindfulness training.

By measuring the simultaneous interplay between the EEG and ECG, we found that APF becomes more correlated with heart activities after 8 weeks of mindfulness meditation. This longitudinal change did not happen during normal rest conditions. We did not find a similar brain-heart connection in the gamma wave band (see Supplementary material). This implies the importance of alpha wave and heart coherence when calculating brain-heart connectivity. Our results demonstrated that APF can be a better EEG measure than alpha power (amplitude) as it is more stable in test-retest reliability (Salinsky et al., 1991; Rocha et al., 2020). The greater between-session variability in EEG power may lead to insignificant findings, as shown in the current results that no significant finding in alpha peak power and its correlation with heart coherence.

Although APF is relatively stable and largely heritable, accumulating evidence indicates that APF can shift slightly during different states, such as sleep, sensorimotor processing or neurological disorders (Uhhaas and Singer, 2006). Mindfulness breathing meditation may modulate the frequency of synchronous neural activity and tune the alpha oscillation to be synchronized with the heart activity. As shown by current results, the instant correlation between APF and Heart coherence became more positive after 8 weeks of MBSR training.

Heart coherence was calculated by the ratio of low and high frequency of heart rate, which partially reflects the balance between the parasympathetic nervous system and sympathetic nervous system (McCraty and Zayas, 2014; McCraty, 2022). Heart coherence is associated with greater levels of emotion regulation and cognitive flexibility. The authors suggested that heart coherence may reflect a state of physiological coherence that is associated with better emotional and cognitive functioning (Shaffer et al., 2014). Heart coherence represents the ordering degree in the oscillation of heart rhythm intervals (Kim et al., 2013). This study found heart coherence is a sensitive marker for meditation states, given its strong correlation with middle-frontal APF and alpha coherence. We did not find a similar correlation between heart coherence and gamma waves. It is plausible that gamma wave is generated by neurons in a firing state and communicating with local cortical areas. Bursts of fast waves, such as gamma and beta waves, play a supportive role in working memory and volitional control.

In contrast, the alpha wave as the slow wave is generated by the thalamo-cortical information interaction, such as feedback loops of excitatory and inhibitory neurons (Dasilva, 1991; van Schie and Bekkering, 2007). This loop enables the cortex to poll information from the thalamus and process it, and then relay the processed information back to the thalamus. Furthermore, the interplay between the thalamus and cortex plays a critical role in a variety of brain functioning, including consciousness and perception (Min et al., 2020). During mindfulness breathing, the practitioner needs to consciously monitor their breath-in and -out. Breathing activity can be either conscious or unconscious. It is assumed that mindful breathing may contribute to increased interaction between brain-heart activities and improve the dynamic oscillatory integration between the peripheral vegetative system (including the cardiovascular system) and the central nervous system. This is vital for individuals to maintain hemostasis, and physical and mental health (Gebber et al., 1999).

Animal studies have found that the rhythmic activity in the respiratory pathway can dynamically modulate the coupling of central oscillators and peripheral targets (Gebber et al., 2004). The discharges of sympathetic nerves generated by central oscillators are around 10 Hz, parallel to the alpha wave band (Gebber et al., 1999). Our results demonstrate that mindfully controlled breathing can cause a slight shift of iAPF in different mind states. This may modulate the coupling of central oscillators and peripheral targets, including cardiac activity, and enhance the cardio-respiratory coordination and, broadly, body-mind connection. Future studies are needed to investigate the role of respiratory activities in connecting cardiac and cerebral activities to determine their potential mediative relationship.

In addition to APF, we found that heart coherence is also correlated with alpha coherence, which is involved in alpha wave generation. A previous study of alpha coherence demonstrated that functional interaction between posterior and anterior brain regions might be involved in generating alpha rhythm during the waking time (Wang et al., 1992). Alpha coherence can be applied to illustrate the functional relationship between different brain regions (Achermann and Borbely, 1998; Cantero et al., 1999; Jorge et al., 2017). The fronto-occipital fasciculi physiologically supports this long-range interaction (Mai et al., 2004). Alpha coherence can discern different arousal levels and is a sensitive index to evaluate various mental states (Cantero et al., 1999). For example, alpha coherence is reduced in patients with Alzheimer’s disease during task that needs memory activity (Hogan et al., 2003). On the other hand, how the sinusoidal oscillations of alpha wave creates coherence among brain regions and its generation mechanism has not been fully illustrated (Miranda de Sá and Infantosi, 2005).

Heartbeat evoked potential reflects the relationship of cardiac electric activity and cerebral electric activity, and it is found to be related to the sense of interoception. In this study, we did not find any longitudinal change in HEP, or any correlation between the HEP and alpha band power, alpha peak frequency, or alpha coherence. We did find great variability of HEP among individuals, and also HEP could be easily contaminated by direct cardiac electric activity. This may contribute to the negative finding of HEP in mindfulness training.

Several limitations are worth noting. Firstly, the number of participants in this brief report is relatively small and no control group was recruited. More participants are in future studies to ensure statistical power after correction for multiple comparisons. The limited sample size and absence of a control group may restrict the generalizability of the findings to broader populations. A second limitation is that although there is a significant increased correlation between cardiac and cerebral activities after mindfulness training, no causal relationship can be made. Additional measurement of breath rate may help explore the causal and mediative relationship between brain, cardiac and respiratory activities.

In sum, 8-week MBSR training can promote brain-heart coherence. Additionally, brain connectivity between two signals provides richer information compared to undirected functional connectivity (Baccala and Sameshima, 2001; Kaminski et al., 2016). The increased alpha rhythm-ECG synchronization at the frontal lobe during MBSR after 8-week training indicates the importance of coherence to maintain an optimal psychophysiological state. It is suggested that the coherence of neurophysiological activity, especially the neurocardiological interplay, plays a vital role in mind and body wellbeing (McCraty et al., 2009). This rhythm-based analysis can also be used to explore other types of meditations which may have different effects on APF and heart coherence.

## Data availability statement

The original contributions presented in this study are included in the article, further inquiries can be directed to the corresponding author.

## Ethics statement

The studies involving human participants were reviewed and approved by the Institutional Review Board (IRB), The University of Hong Kong. The patients/participants provided their written informed consent to participate in this study.

## Author contributions

JG conducted the experiment and prepared the manuscript. RS analyzed the data and experiment design. HL assisted on data analysis, and together with AR and BW, helped prepare the manuscript and shared important ideas. ET and AT helped on statistics and experiment data analysis. HS helped on the experiment design and coordinated the study. All authors contributed to the article and approved the submitted version.
