# Supplementary material for: Increased neurocardiological interplay after mindfulness meditation: a brain oscillation-based approach
Source: Front Hum Neurosci. 2023 Jun 19;17:1008490. doi: 10.3389/fnhum.2023.1008490 (PMC10315629; doi:10.3389/fnhum.2023.1008490)
Supplement: Supplementary file 1 [file Data_Sheet_1.PDF]

Supplementary figures

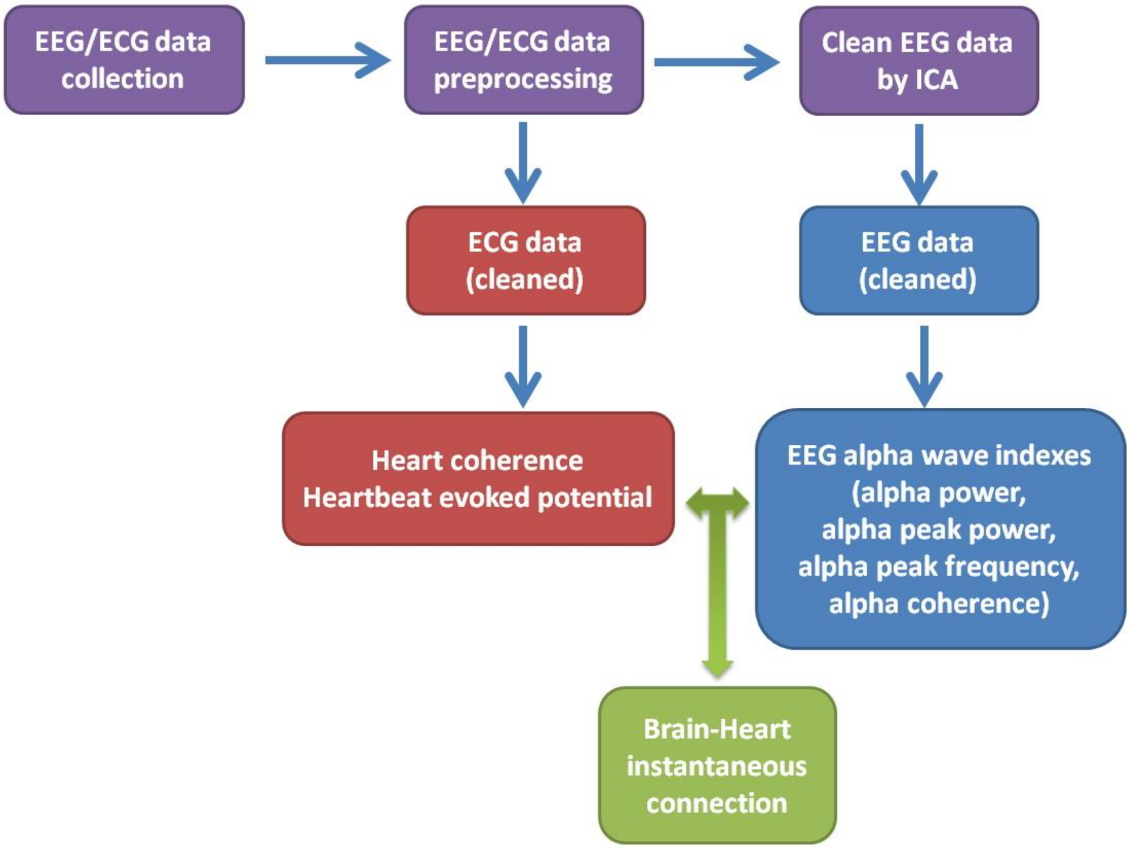

sFigure 1. A flowchart for data analysis.

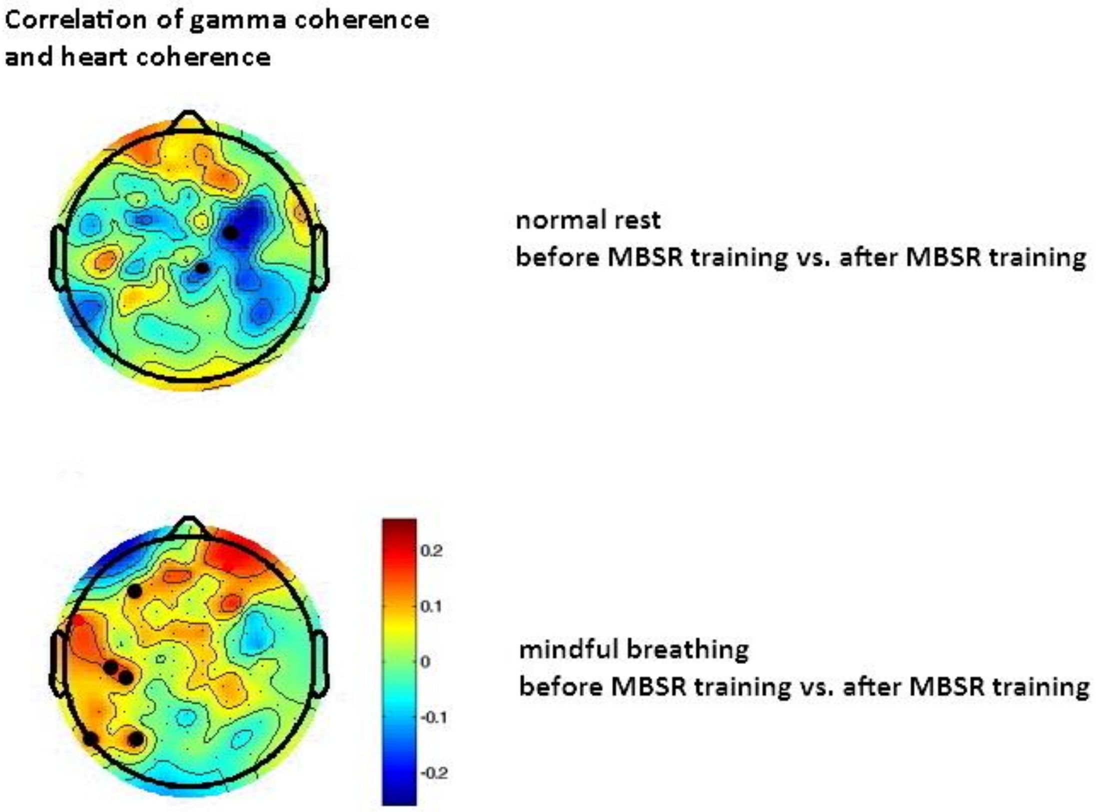

sFigure 2. Maps of brain-heart connections. This connection (*r*-value) was calculated based on correlation of gamma wave coherence and heart coherence. Upper row is showing normal rest condition; lower row is showing the mindful breathing condition. Color represents the *r*-value difference between before and after MBSR training. Dots represent channels with significant difference between before and after MBSR training ($p < 0.05$, uncorrected). Gamma peak frequency was not calculated because gamma wave band (30-45 Hz) has no obvious peak frequency.

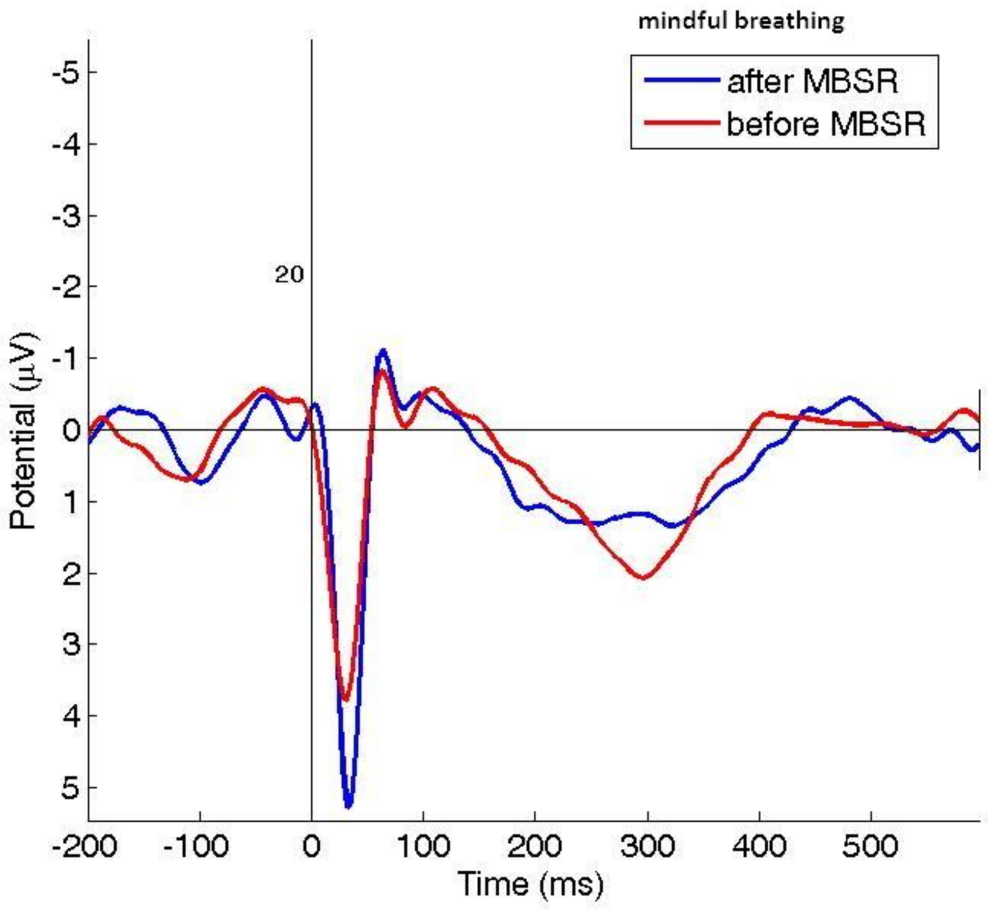

sFigure 3. A channel (no. 20 of NeuroScan128-channels Quik-Cap) at the leftmost edge of the occipital area showing the averaged heartbeat evoked potential (HEP) of mindful breathing before and after MBSR intervention. No significant difference was found.

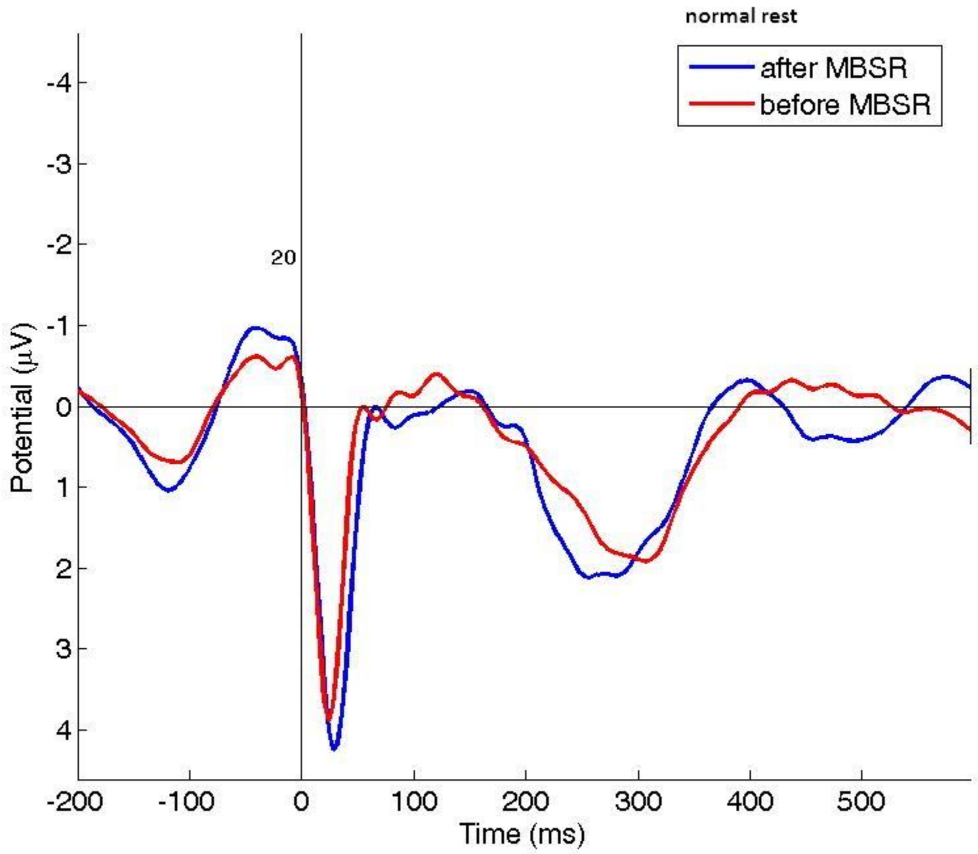

sFigure 4. A channel (no. 20) at the leftmost edge of occipital area showing the averaged heartbeat evoked potential of normal rest before and after MBSR intervention. No significant difference was found.

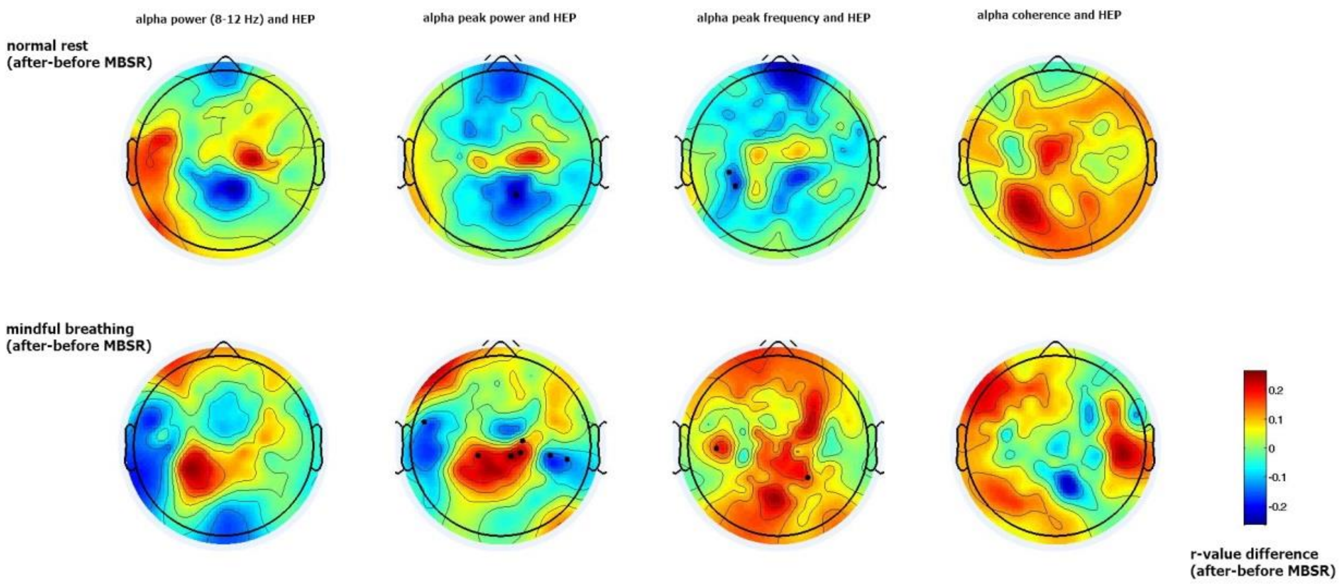

sFigure 5. Maps of alpha wave indexes and heartbeat evoked potential (HEP) correlation. HEP was taken from a channel (no. 20) at bottom left of occipital area. Upper row: normal rest condition; lower row: mindful breathing condition. Color represents the *r*-value difference between before and after MBSR training. Dots represent channels with significant difference between before and after MBSR training ($p < 0.05$, uncorrected).

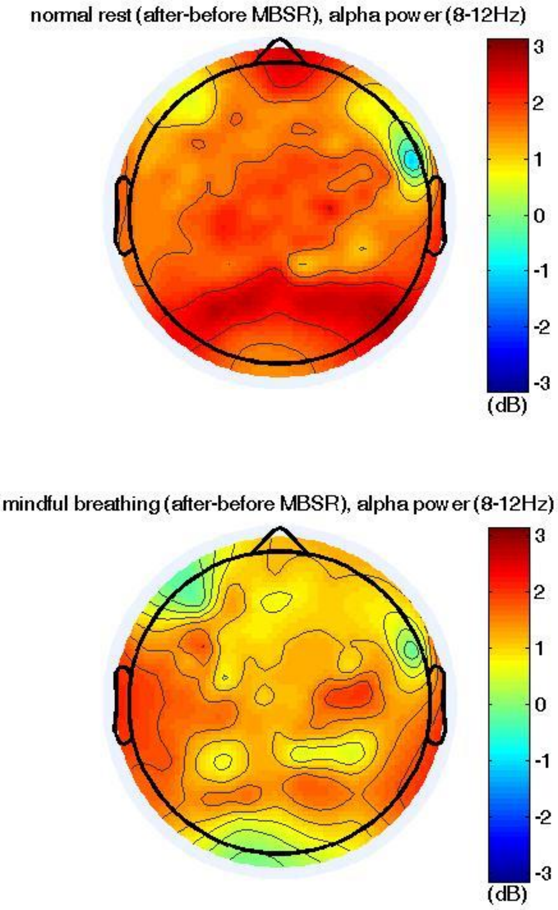

sFigure 6. Maps of alpha power difference. No significant difference was found.

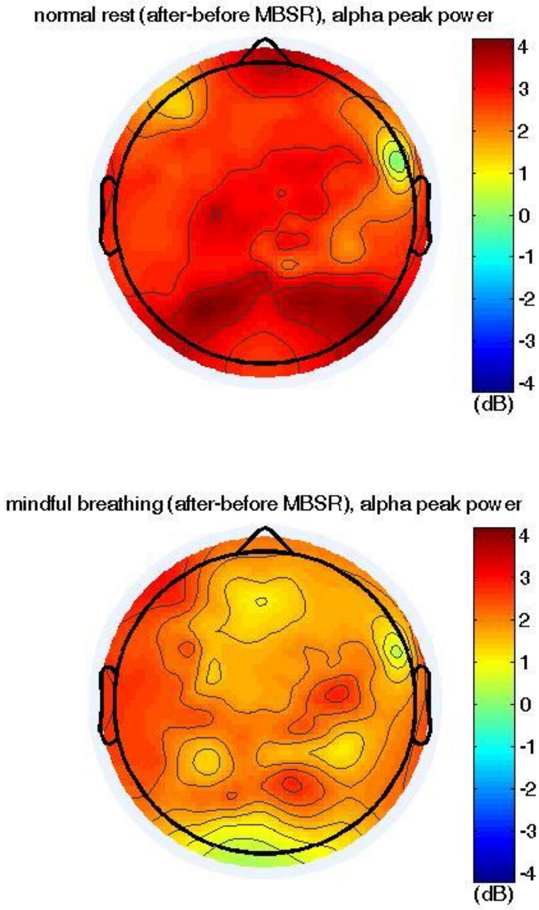

sFigure 7. Maps of alpha peak power difference. No significant difference was found.

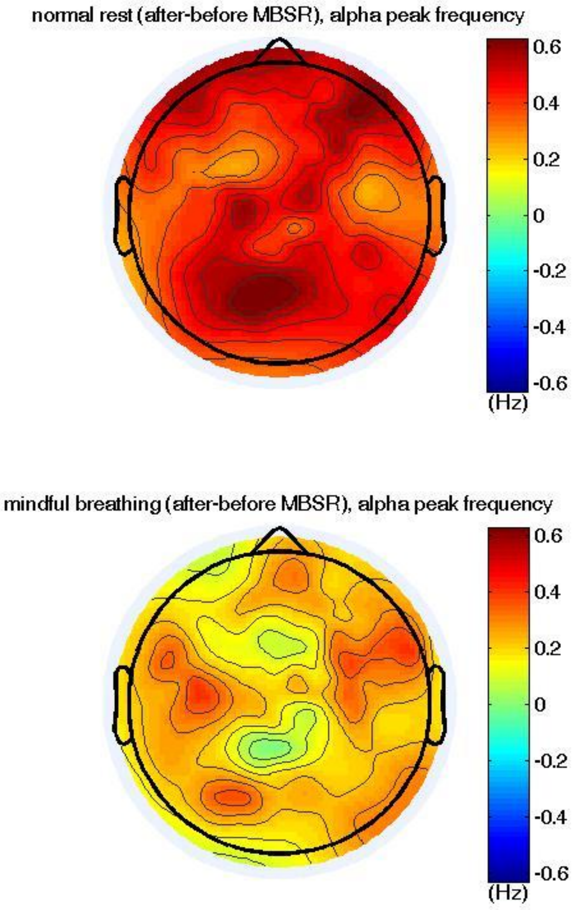

sFigure 8. Maps of alpha peak frequency difference. No significant difference was found.

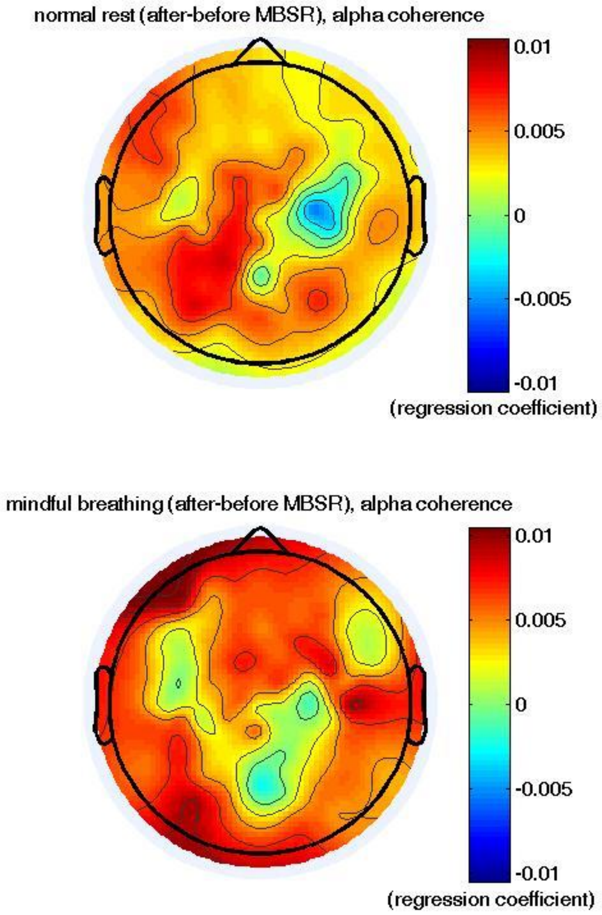

sFigure 9. Maps of alpha coherence difference. No significant difference was found.

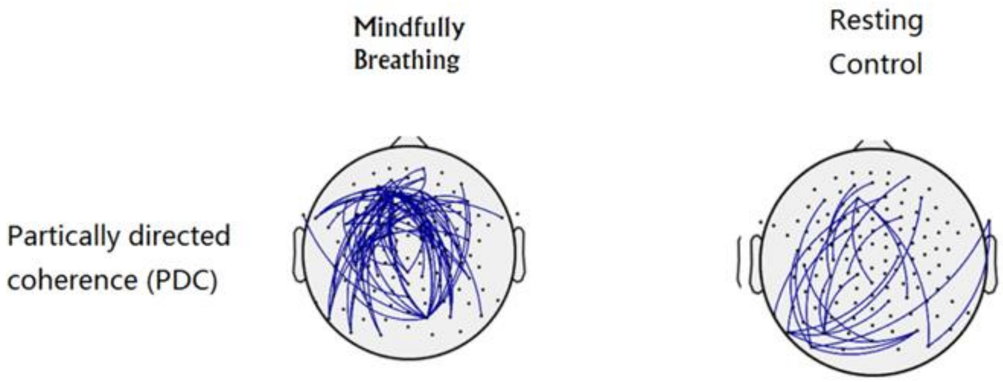

sFigure 10. After the 8-week mindfulness training, PDC was increased during mindful breathing (left map) when comparing before and after MBSR intervention. Similar changes were found during normal rest (right map) but not as prevalent. Each line indicates a significant correlation between two channels ($p < 0.01$, uncorrected). Blue represent an increase of PDC, red represent a decrease of PDC (none was found). Effective connectivity measures the synchronous oscillation between different brain regions. Current data shows an increased brain effective connectivity after MBSR training in terms of partial directed coherence (PDC). PDC is a frequency-domain approach to estimating the direction of information flow between multivariate signals. The estimation is based on the decomposition of partial coherences from multivariate auto-regressive models (Baccala and Sameshima, 2001). Increased PDC after MBSR implies stronger coherent activities among the central regions during mindful breathing. After eight weeks of training, the participant may have greater capability to inhibit the wandering mind and better control of mindful breathing (Jovanovic et al., 2013). Previous studies have found that mindfulness training can alter the brain connectivity in neural networks related to mind wandering, attention, executive function and emotional reactivity (Vishnubhotla et al., 2021;Melis et al., 2022). Increased connectivity in the central-frontal regions could indicate that MBSR can enhance the default mode network-prefrontal cortex connectivity and thus focus attention (Melis et al., 2022).

Supplementary tables

EEG channel	M2 *r*-value mean	*SD*	M1 *r*-value mean	*SD*	*r*-value difference	*t*(10)	*p*	**Cohen's d**
5	0.037	0.137	-0.113	0.158	0.150	2.45	0.034	0.74
7	0.030	0.101	-0.120	0.189	0.150	2.59	0.027	0.78
8	0.061	0.114	-0.170	0.207	0.231	3.98	0.003	1.20
13	0.083	0.133	-0.144	0.238	0.227	2.95	0.015	0.89
15	0.046	0.110	-0.171	0.160	0.217	3.03	0.013	0.91
16	0.078	0.172	-0.086	0.127	0.163	2.29	0.045	0.69
17	0.037	0.155	-0.180	0.147	0.216	3.15	0.010	0.95
18	0.047	0.143	-0.187	0.151	0.234	4.07	0.002	1.23
19	0.091	0.155	-0.149	0.198	0.240	3.73	0.004	1.12
20	0.085	0.150	-0.160	0.194	0.245	4.27	0.002	1.29
21	0.058	0.179	-0.216	0.180	0.274	4.74	0.001	1.43
23	0.133	0.151	-0.171	0.234	0.304	3.35	0.007	1.01
24	0.117	0.163	-0.091	0.131	0.209	2.89	0.016	0.87
28	0.048	0.100	-0.145	0.231	0.193	2.91	0.015	0.88
30	0.134	0.142	-0.038	0.240	0.172	2.24	0.049	0.68
33	0.048	0.111	-0.147	0.243	0.195	2.57	0.028	0.78
34	0.042	0.132	-0.154	0.222	0.197	2.98	0.014	0.90
35	0.074	0.161	-0.200	0.175	0.274	3.35	0.007	1.01
36	0.057	0.189	-0.157	0.217	0.214	2.74	0.021	0.83
37	0.088	0.193	-0.115	0.264	0.204	2.51	0.031	0.76
39	0.113	0.228	-0.091	0.172	0.204	2.46	0.033	0.74
42	0.067	0.183	-0.185	0.208	0.252	3.47	0.006	1.05
43	0.073	0.188	-0.204	0.195	0.277	3.55	0.005	1.07
45	0.066	0.177	-0.132	0.221	0.198	2.23	0.050	0.67
47	0.148	0.208	-0.032	0.195	0.180	2.84	0.018	0.86
51	0.029	0.201	-0.182	0.161	0.211	2.50	0.032	0.75
52	0.079	0.172	-0.186	0.147	0.265	3.56	0.005	1.07
53	0.096	0.166	-0.162	0.195	0.258	3.28	0.008	0.99
54	0.097	0.159	-0.165	0.235	0.262	2.98	0.014	0.90
55	0.100	0.139	-0.134	0.240	0.234	2.91	0.016	0.88
58	0.154	0.166	-0.098	0.294	0.252	2.39	0.038	0.72
59	0.139	0.133	-0.151	0.256	0.290	3.60	0.005	1.09
60	0.098	0.175	-0.155	0.241	0.253	2.75	0.020	0.83

61	0.099	0.195	-0.182	0.211	0.280	3.03	0.013	0.91
62	0.110	0.178	-0.170	0.181	0.280	3.77	0.004	1.14
70	0.104	0.155	-0.186	0.203	0.290	3.32	0.008	1.00
79	0.104	0.191	-0.169	0.220	0.273	3.40	0.007	1.03
80	0.093	0.175	-0.163	0.240	0.257	2.86	0.017	0.86
81	0.149	0.166	-0.134	0.245	0.283	3.92	0.003	1.18
82	0.129	0.139	-0.111	0.272	0.240	2.89	0.016	0.87
83	0.139	0.132	-0.107	0.248	0.246	3.06	0.012	0.92
87	0.117	0.161	-0.091	0.234	0.209	2.51	0.031	0.76
88	0.148	0.185	-0.113	0.244	0.261	3.24	0.009	0.98
89	0.122	0.195	-0.107	0.260	0.229	2.51	0.031	0.76
90	0.120	0.163	-0.097	0.221	0.217	2.70	0.022	0.81
98	0.068	0.176	-0.188	0.196	0.255	3.11	0.011	0.94
99	0.040	0.196	-0.178	0.183	0.218	2.45	0.034	0.74
103	0.047	0.185	-0.134	0.181	0.181	2.23	0.050	0.67
106	0.091	0.129	-0.080	0.239	0.172	2.38	0.038	0.72
107	0.110	0.121	-0.108	0.232	0.218	2.79	0.019	0.84
113	0.064	0.113	-0.097	0.214	0.161	2.28	0.046	0.69
114	0.028	0.141	-0.105	0.194	0.133	2.59	0.027	0.78
115	0.055	0.129	-0.116	0.192	0.170	3.55	0.005	1.07
117	0.063	0.146	-0.136	0.219	0.199	2.64	0.025	0.80
118	0.054	0.150	-0.131	0.215	0.185	2.75	0.020	0.83
119	0.047	0.145	-0.164	0.194	0.211	3.89	0.003	1.17
120	0.088	0.137	-0.134	0.180	0.221	3.39	0.007	1.02
121	0.106	0.131	-0.073	0.183	0.179	2.74	0.021	0.83
122	0.088	0.120	-0.109	0.201	0.196	2.74	0.021	0.83
124	0.135	0.226	-0.057	0.196	0.192	2.40	0.037	0.72

sTable 1. Statistic details for each channel with significant difference shown in Figure 1 (bottom left). Paired-sample t-test was used. M2: mindful breathing after MBSR training; M1: mindful breathing before MBSR training; *r*-value is the correlation coefficient between alpha peak frequency and heart coherence.

EEG channel	M2 *r*-value mean	*SD*	M1 *r*-value mean	*SD*	*r*-value difference	*t*(10)	*p*	**Cohen's d**
34	0.102	0.191	-0.050	0.242	0.151	2.25	0.048	0.68
35	0.137	0.194	-0.091	0.230	0.229	4.39	0.001	1.32
36	0.098	0.184	-0.062	0.218	0.159	3.07	0.012	0.93
41	0.127	0.234	-0.059	0.212	0.187	2.34	0.041	0.71
42	0.150	0.215	-0.051	0.260	0.201	2.68	0.023	0.81
44	0.160	0.239	-0.031	0.265	0.191	2.62	0.026	0.79
51	0.092	0.224	-0.053	0.238	0.145	2.51	0.031	0.76
52	0.144	0.207	-0.095	0.234	0.239	4.18	0.002	1.26
53	0.126	0.208	-0.105	0.240	0.230	3.56	0.005	1.07
54	0.101	0.204	-0.100	0.221	0.202	3.55	0.005	1.07
60	0.089	0.220	-0.083	0.236	0.173	2.78	0.019	0.84
61	0.102	0.215	-0.107	0.252	0.209	3.38	0.007	1.02
62	0.138	0.209	-0.099	0.255	0.237	3.88	0.003	1.17
63	0.117	0.218	-0.077	0.254	0.194	3.19	0.010	0.96
67	0.078	0.233	-0.139	0.124	0.217	2.42	0.036	0.73
78	0.115	0.247	-0.075	0.281	0.190	2.63	0.025	0.79
79	0.107	0.254	-0.073	0.276	0.180	2.53	0.030	0.76
80	0.099	0.252	-0.065	0.247	0.164	2.46	0.034	0.74
120	0.116	0.296	-0.013	0.290	0.129	2.26	0.048	0.68
123	0.113	0.174	-0.072	0.220	0.185	2.91	0.016	0.88
124	0.130	0.171	-0.104	0.148	0.234	3.94	0.003	1.19
125	0.098	0.160	-0.071	0.182	0.169	2.66	0.024	0.80
126	0.080	0.230	-0.090	0.232	0.170	2.48	0.032	0.75
128	0.123	0.281	-0.051	0.259	0.174	2.57	0.028	0.78

sTable 2. Statistic details for each channel with significant difference shown in Figure 1 (bottom right). Paired-sample t-test was used. M2: mindful breathing after MBSR training; M1: mindful breathing before MBSR training. *r*-value is the correlation coefficient between alpha coherence and heart coherence.

Pair	channelA	channelB	M2 PDC	M1 PDC	$t(10)$	p	Cohen's d
1	67	6	0.0640	0.0289	3.17	0.0099	0.96
2	82	6	0.0729	0.0305	3.48	0.0059	1.05
3	61	8	0.0804	0.0428	3.25	0.0087	0.98
4	117	8	0.0825	0.0357	3.25	0.0087	0.98
5	125	8	0.0715	0.0267	3.41	0.0067	1.03
6	7	12	0.1010	0.0425	3.67	0.0043	1.11
7	117	13	0.0942	0.0406	3.21	0.0094	0.97
8	29	15	0.0707	0.0225	3.59	0.0049	1.08
9	41	15	0.0675	0.0290	3.34	0.0075	1.01
10	49	15	0.0761	0.0251	3.30	0.0080	1.00
11	50	15	0.0695	0.0271	3.23	0.0090	0.98
12	56	15	0.0765	0.0225	3.37	0.0072	1.01
13	71	15	0.0659	0.0202	3.32	0.0077	1.00
14	79	15	0.0676	0.0193	3.29	0.0081	0.99
15	91	15	0.0763	0.0251	3.41	0.0067	1.03
16	113	15	0.0852	0.0318	3.24	0.0088	0.98
17	121	15	0.0874	0.0296	3.80	0.0035	1.15
18	13	16	0.0702	0.0273	3.34	0.0075	1.01
19	14	16	0.0727	0.0168	3.89	0.0030	1.17
20	82	16	0.0817	0.0259	3.24	0.0089	0.98
21	112	16	0.0636	0.0207	3.22	0.0092	0.97
22	18	33	0.0934	0.0430	3.56	0.0052	1.07
23	44	33	0.0851	0.0387	3.30	0.0080	0.99
24	55	33	0.0896	0.0371	3.43	0.0065	1.03
25	59	33	0.0850	0.0324	3.84	0.0032	1.16
26	65	33	0.0961	0.0406	3.41	0.0066	1.03
27	66	33	0.0915	0.0328	3.69	0.0042	1.11
28	102	33	0.0847	0.0379	3.32	0.0078	1.00
29	122	33	0.0789	0.0417	3.18	0.0099	0.96
30	2	54	0.0848	0.0373	3.70	0.0041	1.12
31	55	54	0.0837	0.0397	3.41	0.0066	1.03
32	66	54	0.0851	0.0406	3.28	0.0083	0.99
33	71	54	0.0922	0.0464	3.43	0.0064	1.03
34	92	54	0.0841	0.0399	3.77	0.0036	1.14
35	97	54	0.0910	0.0460	3.35	0.0074	1.01
36	2	55	0.0839	0.0359	3.99	0.0026	1.20
37	3	55	0.0849	0.0388	3.23	0.0090	0.97

38	18	55	0.0917	0.0390	4.69	0.0009	1.41
39	20	55	0.0870	0.0380	3.34	0.0075	1.01
40	23	55	0.0754	0.0341	3.25	0.0087	0.98
41	29	55	0.0865	0.0400	3.41	0.0066	1.03
42	32	55	0.0858	0.0383	3.17	0.0100	0.96
43	39	55	0.0829	0.0371	3.26	0.0086	0.98
44	43	55	0.0765	0.0351	3.67	0.0043	1.11
45	44	55	0.0849	0.0332	4.06	0.0023	1.22
46	51	55	0.0819	0.0319	3.41	0.0067	1.03
47	53	55	0.0846	0.0321	3.27	0.0084	0.99
48	65	55	0.0906	0.0349	4.45	0.0012	1.34
49	66	55	0.0834	0.0300	3.68	0.0042	1.11
50	71	55	0.1001	0.0341	4.04	0.0024	1.22
51	74	55	0.0909	0.0337	4.19	0.0019	1.26
52	83	55	0.0846	0.0361	3.76	0.0037	1.13
53	91	55	0.0842	0.0324	3.24	0.0088	0.98
54	92	55	0.0847	0.0337	3.83	0.0033	1.15
55	95	55	0.0953	0.0381	3.90	0.0030	1.18
56	97	55	0.0979	0.0350	3.47	0.0061	1.05
57	99	55	0.0921	0.0368	3.82	0.0034	1.15
58	102	55	0.0878	0.0354	3.55	0.0052	1.07
59	106	55	0.0915	0.0332	3.45	0.0062	1.04
60	115	55	0.0774	0.0348	3.37	0.0071	1.02
61	116	55	0.0874	0.0328	3.74	0.0039	1.13
62	71	56	0.0937	0.0434	3.23	0.0090	0.97
63	2	60	0.0816	0.0356	3.97	0.0026	1.20
64	23	60	0.0722	0.0373	3.23	0.0090	0.97
65	55	60	0.0798	0.0401	3.18	0.0098	0.96
66	71	60	0.0899	0.0430	3.20	0.0094	0.97
67	92	60	0.0800	0.0384	3.38	0.0070	1.02
68	97	60	0.0884	0.0404	3.33	0.0076	1.00
69	1	71	0.1005	0.0397	3.67	0.0043	1.11
70	22	71	0.0892	0.0271	3.38	0.0070	1.02
71	26	71	0.0974	0.0357	3.19	0.0097	0.96
72	51	71	0.1087	0.0340	3.26	0.0086	0.98
73	95	71	0.0916	0.0347	3.21	0.0093	0.97
74	106	71	0.0815	0.0296	3.20	0.0094	0.97
75	109	71	0.0879	0.0326	3.48	0.0059	1.05

76	112	71	0.0980	0.0269	3.53	0.0055	1.06
77	2	80	0.0711	0.0312	3.19	0.0097	0.96
78	20	80	0.0854	0.0477	3.44	0.0064	1.04
79	43	80	0.0746	0.0390	3.87	0.0031	1.17
80	112	80	0.0720	0.0357	3.27	0.0084	0.99
81	2	81	0.0839	0.0341	3.85	0.0032	1.16
82	20	81	0.0912	0.0483	3.43	0.0064	1.03
83	43	81	0.0868	0.0437	3.25	0.0087	0.98
84	65	81	0.0838	0.0460	3.28	0.0083	0.99
85	71	81	0.0919	0.0456	3.35	0.0073	1.01
86	83	81	0.0860	0.0450	3.18	0.0099	0.96
87	106	81	0.0918	0.0426	3.29	0.0081	0.99
88	116	81	0.0842	0.0416	3.22	0.0092	0.97

sTable3. Statistic details for each pair of channels with significant difference shown in sFigure 10 (left, $p < .01$ uncorrected). Paired-sample t-test was used. M2: mindful breathing after MBSR training; M1: mindful breathing before MBSR training; PDC: partial directed coherence.

Pair	channelA	channelB	R2 PDC	R1 PDC	*t*(10)	*p*	Cohen's d
1	81	19	0.0667	0.0379	3.50	0.0057	1.06
2	53	20	0.1056	0.0496	3.25	0.0086	0.98
3	73	20	0.0875	0.0391	3.25	0.0087	0.98
4	92	20	0.1011	0.0395	3.23	0.0090	0.97
5	97	20	0.1068	0.0301	4.25	0.0017	1.28
6	29	21	0.1016	0.0443	3.56	0.0052	1.07
7	97	21	0.1016	0.0325	4.23	0.0017	1.28
8	59	27	0.0725	0.0240	3.25	0.0087	0.98
9	24	42	0.1100	0.0486	3.32	0.0077	1.00
10	49	42	0.1060	0.0504	3.27	0.0084	0.99
11	86	42	0.1047	0.0435	3.82	0.0034	1.15
12	108	42	0.1261	0.0473	3.66	0.0044	1.10
13	4	43	0.1086	0.0423	3.84	0.0032	1.16
14	34	43	0.1114	0.0454	3.26	0.0085	0.98
15	97	43	0.1142	0.0438	3.31	0.0079	1.00
16	25	55	0.0601	0.0281	3.20	0.0096	0.96
17	65	55	0.0603	0.0307	4.31	0.0015	1.30
18	95	55	0.0751	0.0301	3.17	0.0099	0.96
19	98	55	0.0612	0.0277	3.42	0.0066	1.03
20	24	56	0.0643	0.0197	3.20	0.0095	0.96
21	6	69	0.1123	0.0441	3.29	0.0082	0.99
22	38	89	0.0491	0.0298	3.22	0.0091	0.97
23	43	124	0.0772	0.0385	3.27	0.0084	0.99
24	98	124	0.0837	0.0407	3.37	0.0072	1.02

sTable4. Statistic details for each pair of channels with significant difference shown in sFigure 10 (right, $p < .01$ uncorrected). Paired-sample t-test was used. R2: normal rest after MBSR training; R1: normal rest before MBSR training; PDC: partial directed coherence.
